# Taking back control of the Data. Developing an all in one System to monitor training post quality and provide trainer feedback

**DOI:** 10.1192/j.eurpsy.2022.2199

**Published:** 2022-09-01

**Authors:** J. Mudunkotuwe, M. Schmidt, A. Khan, T. Sahlsberg

**Affiliations:** 1 Surrey and Borders NHS Foundation Trust, Neurodevelopmental Service, Epsom, United Kingdom; 2 Health Education England, Kent Surrey And Sussex, Psychiatry, Redhill, United Kingdom; 3 Surrey and Borders NHS foundation Trust, Medical Education, Epsom, United Kingdom; 4 ReportGorilla, It Consulting, Surrey, United Kingdom

**Keywords:** Supervisor, appraisal, education, Multi source feedback

## Abstract

**Introduction:**

We will present experience developing a system for monitoring training placements in psychiatry and community paediatrics, and how this was expanded to provide an automated anonymised MSF for trainers for annual appraisal and will identify trainers in need of additional support and other post/training programme issues. The session will be of interest to educators and medical education leads with practical tips and lessons learnt over the last 8 years since the system was first developed.

**Objectives:**

The system was also used to identify trainers in need of additional support and other post/training programme issues.

**Methods:**

We used an electronic system to gain the infromation as stated in the introduction.

**Results:**

Over the last 8 years we have collected data using this system. the results for our trust will be displayed annoymously but the system is the ficus of this presenation.

**Conclusions:**

The advantages of the system are that it runs throughout the year (so covers each post and placement), has high trainee response rates, has no selection bias (compared with some other MSF systems) and the results are embedded within local quality systems and individual consultant appraisals. The data that the system collects can help provide robust evidence when investigating concerns that might only arise periodically (for example through the annual GMC trainee survey in the UK). We believe that this system will be applicable for doctors providing training in other countries and empowers the improvement of psychiatric training for the profession.

**Disclosure:**

No significant relationships.

